# Maximising the Potential of Reactive Carbon Support with Cobalt Active Phase for the Oxygen Evolution Reaction

**DOI:** 10.3390/molecules30071522

**Published:** 2025-03-29

**Authors:** Termeh Darvishzad, Paweł Stelmachowski

**Affiliations:** Faculty of Chemistry, Jagiellonian University, Gronostajowa 2, 30-387 Krakow, Poland

**Keywords:** renewable energy, surface modification, plasma oxidation, carbon composites, oxygen evolution reaction, hydrogen production

## Abstract

A growing interest in novel noble metal-free electrocatalysts is fuelled by the pressing need to overcome the drastic demand for sustainable energy sources. To this end, the oxygen evolution reaction (OER) utilising transition metal oxide–carbon composites in alkaline media is considered a robust technology. In many such systems, carbon is used as a conductive additive or support, and the interactions between carbon support materials and the active phase affect the efficiency of the electrocatalyst. Cobalt forms some of the most active and stable electrocatalysts for OER. In carbon-supported systems, the dispersion of the cobalt phase on the carbon surface is a key factor in influencing the catalyst activity in water-splitting reactions. In this study, a low-temperature plasma treatment is used to boost the efficiency of the cobalt active phase by functionalising the carbon support with various oxygen groups. We used a simple deposition–precipitation method to obtain cobalt hydroxide active phase over graphene nanoparticles. The activation of graphene nanoparticles with oxygen plasma allowed us to obtain a catalyst that showed only 317 mV@10 mA·cm^−2^. More importantly, in the series of plasma-activated samples, the OER activity was very high in a range of cobalt phase loadings, yielding a material with 2.4 wt.% of cobalt and an overpotential of only 327 mV@10 mA·cm^−2^. The results indicate that plasma activation of GNP support maximises the usage of the transition metal active phase, which allows for an improvement in area-normalised and a dramatic improvement in the mass-normalised OER electrocatalytic activity.

## 1. Introduction

Due to the global demand for renewable energy sources, many researchers worldwide are motivated to investigate advanced electrocatalysts, including oxygen evolution reaction (OER) catalysts [1,2,3]. Due to its sluggish kinetics, this anodic half-reaction is considered a bottleneck for energy conversion and storage systems [4]. Carbon-based systems—promising composite materials—have been applied metal-free and doped with non-precious transition metals in many studies [5,6]. Among carbon materials, graphene nanoplatelets (GNP) modified with transition-metal oxides have gained attraction in the energy storage field [7]. GNP studies show excellent electrical conductivity, flexibility, and large specific surface area [8]. GNP’s porous structure and acidic and basic sites make them suitable substances with promising catalytic properties [8]. GNP-based composites are attractive materials for preparing catalysts for water-splitting reactions and have applications in other electrochemical approaches such as batteries, supercapacitors, and sensors [9].

The most active OER electrocatalysts are usually based on noble metals such as platinum, iridium and ruthenium [10,11]. To overcome the availability limitations of noble metals, transition metals like cobalt and nickel are receiving more and more attention, even on industrial scales [12,13,14]. They have the advantages of scalable synthesis, high efficiency, corrosion resistance, and promising catalytic activity [15]. Thanks to the various oxidation states of cobalt, the application of cobalt oxide/hydroxide-modified carbons, including GNPs, is continuously popular in water-splitting reactions [16]. Cobalt spinel (Co_3_O_4_) consists of two electronically mixed valences of Co^2+^ and Co^3+^, which makes it a promising candidate for use in OER technology and Li-O_2_ batteries. Co^2+^ cations facilitate the formation of cobalt oxyhydroxide (CoOOH) intermediate, the key factor in the OER catalytic process. Co(OH)_2_ is a gateway substance for transforming CoOOH and Co_3_O_4_. CoOOH can be converted to Co(OH)_2_ by calcination at a modest temperature. Thermal decomposition of Co(OH)_2_ and CoOOH is a common method of Co_3_O_4_ production [17,18].

Carbon surface modification is usually necessary to improve its interactions with the catalytically active phase. Various methods have been used to functionalise carbon surfaces, such as wet oxidation by strong acids, which sometimes accompanies partial deterioration of the addressed substance [6]. Our group recently discovered the speciation of oxygen functional groups on carbon surfaces with a positive correlation between carboxylic groups and the electrocatalytic activity of their researched catalysts. We successfully showed that using plasma oxidation can modify the carbon support surface without damaging the structure of carbon. In contrast to invasive surface modification techniques, plasma oxidation is a mild method that sufficiently increases the surface concentration of oxygen groups. Consequently, it enhances the catalytic properties of the modified composite and makes it more suitable for use in OER [8,19,20].

In this research, we studied different oxidation procedures of GNP as supports for the Co(OH)_2_ active phase. We used plasma oxidation to functionalise the surface of GNP with oxygen groups. Since plasma treatment activates the carbon material and introduces functional groups [20], we compared freshly treated samples with aged samples. As references, we used non-oxidised GNP and ammonium persulfate-oxidised GNP. This study aimed to determine how different surface oxidation states of GNP influence the activity of the cobalt active phase in the oxygen evolution reaction.

## 2. Results and Discussions

**Figure 1:**
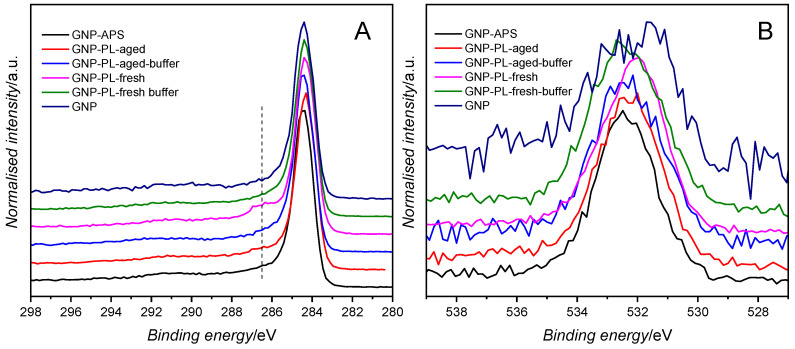
XPS spectra of GNP support oxidised using different procedures. (**A**) C 1s narrow range; (**B**) O 1s narrow range.

The plasma pretreatment of the GNP resulted in varied oxidation levels of the carbon support. The XPS analysis shows that the unmodified graphene nanoplatelets contain only a small fraction of surface oxygen, 2.2 at.%. Wet oxidation with APS increases the surface oxygen content to 6.1 at.%. Plasma oxidation leads to a substantial increase in oxygen content up to 11.6 at.% (GNP-PL-fresh), but sample ageing in the air atmosphere for 14 days decreases surface oxygen to 8.0 at.% (GNP-PL-aged). The decrease in the oxygen functional groups over time has been ascribed to oxygen species surface diffusion, recombination into H_2_O, and subsequent desorption [21]. To mitigate this detrimental effect and to stabilise the samples, a simple water immersion and drying technique can be applied to obtain stable surface functionalities [8,20]. The XPS analysis of selected samples is presented in Table S1. Then, the GNP samples were subjected to acetate buffer, which was used to deposit the cobalt active phase. This procedure aimed to evaluate the effect of the environment of the cobalt phase deposition on the surface state of oxygen groups on GNP [8,20]. Plasma-treated GNP washed with acetate buffer, rinsed with deionised water and dried decreased oxygen content for fresh and aged GNP to 8.1 and 5.6 at.%, respectively. Figure 1A shows normalised C 1s spectra of the GNP supports used in this study. Notably, a pronounced band at 286.6 eV due to C=O species is visible for the GNP-PL-fresh sample. Similarly, the O 1s band is shifted about 0.5 eV for this sample towards the lower binding energies, indicating a higher abundance of oxygen species in carbonyl, lactone and carboxylic groups.

Our previous studies examined the impact of oxygen functional groups on oxidised carbons. We discovered that, regardless of the total surface oxygen content, the relative abundance of carboxylic-type oxygen groups plays a key role in determining the dispersion and activity of the cobalt oxide–carbon composite catalyst. Oxidative pretreatment does not directly affect the amount of the deposited active phase when employing equilibrium adsorption–precipitation for cobalt oxide deposition. Our findings indicate that the reactivity in the oxygen evolution reaction is influenced by the active phase’s quantity and dispersion; samples with the most dispersed cobalt oxide demonstrate the highest activity [19].

**Figure 2:**
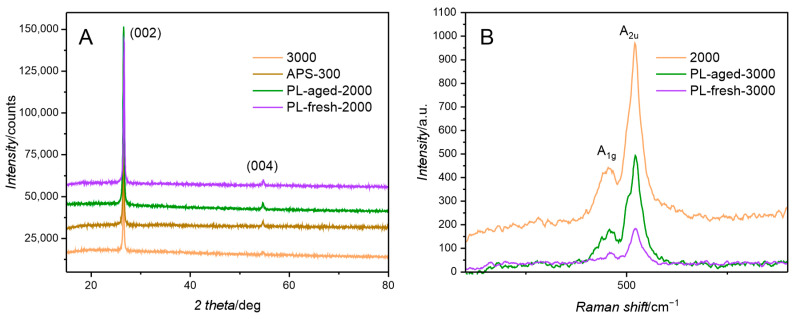
(**A**) X-ray diffraction patterns and (**B**) Raman spectra of selected composite catalysts.

**Figure 3:**
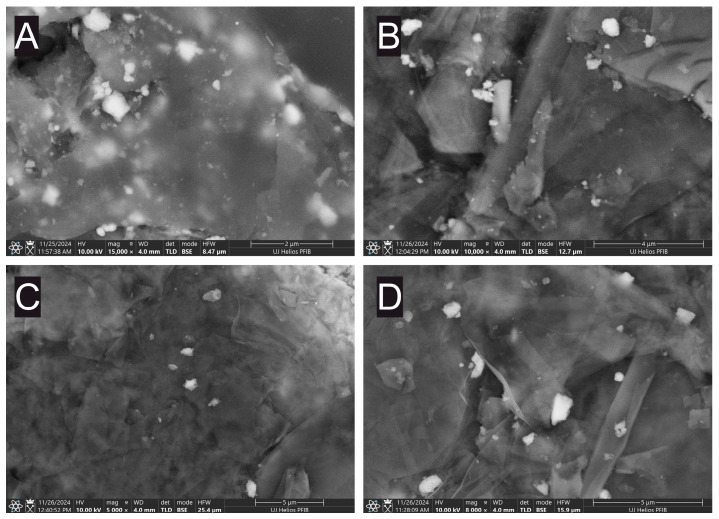
SEM images of selected samples recorded in BSE mode. (**A**) 2000, (**B**) APS-1500, (**C**) PL-aged-2000, and (**D**) PL-fresh-1500.

The adopted simple deposition–precipitation of the cobalt hydroxide resulted in the formation of composite catalysts with increasing cobalt content, as summarised in Table S2 (XPS) and Table S3 (XRF). For simplicity, the samples will be named according to the concentration of the Co^2+^ ions in the precursor solution, omitting the support GNP designation. XRD patterns show reflections of 26.6 and 54.7 2Θ values typical of graphitic carbon structures, originating from (002) and (004) lattice planes, respectively (Figure 2A). Despite the high loading of the cobalt phase, no reflections from its crystalline forms are visible. It suggests that cobalt is present in an amorphous state. This conclusion is supported by the Raman spectra, which yield bands characteristic of specific atomic arrangements rather than long-range ordering. The bands at 454 and 521 cm^−1^ are attributed to the Co(OH)_2_ structure (Figure 2B) [17]. Thus, the active phase in composite materials obtained is amorphous cobalt hydroxide. SEM imaging was used to study the morphology of selected samples. Representative images are presented in Figure 3 and Figure S1 in the SI. Visible cobalt hydroxide particles are polydisperse with various agglomerations ranging from hundreds of nanometres to a few micrometres, regardless of the GNP pretreatment method.

**Figure 4:**
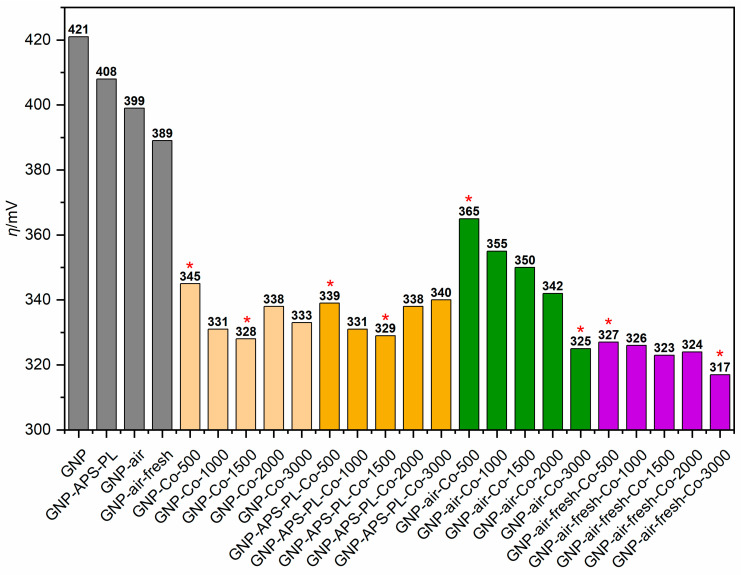
Overpotentials @10 mA·cm^−2^, created by four series of cobalt-doped catalysts in OER, compared with their references, without cobalt. Grey colour—reference GNP (without cobalt phase); light beige—unmodified GNP with cobalt phase; light brown—ammonium persulfate-oxidised GNP with cobalt phase; green—plasma-modified and aged GNP with cobalt phase; purple—freshly plasma-modified GNP with cobalt phase. The red asterisk denotes samples subject to a deeper analysis.

Based on CA measurements (Figure S2), the electrocatalytic activity was evaluated as overpotentials (*ƞ*) at the current density of 10 mA·cm^−2^. The reference GNP and oxidised GNP activity, indicated by the grey colour in Figure 4, follows the surface oxygen content determined with XPS. It is well-established that the surface functionalisation of carbon materials improves the OER electrocatalytic activity [22]. Catalysts from all four cobalt-doped series substantially decreased *ƞ* compared to all reference GNP. Interestingly, the activity trends in each series differ. For the non-modified and APS-oxidised supports, the optimum activity is observed for the samples obtained with the cobalt concentration of 1500 mg·dm^−3^. The activity in the plasma-treated and aged series increases monotonously. However, deposition of the cobalt phase over freshly plasma-treated GNP results in the highest overall activities, including the sample with the lowest cobalt loading. The samples marked with a red asterisk were selected for further analysis.

**Figure 5:**
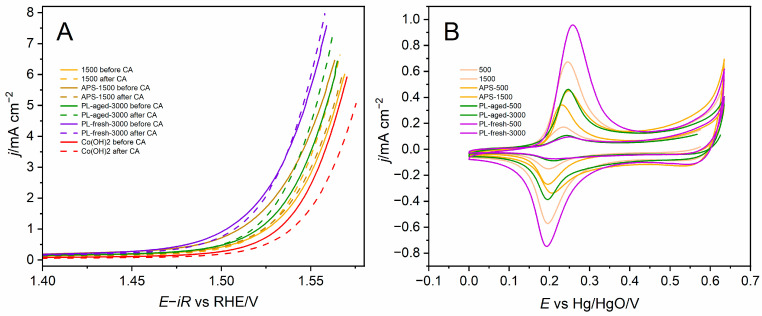
(**A**) iR-corrected LSVs recorded before and after CA, for cobalt hydroxide and the best catalyst from each series. (**B**) CVs recorded before the CA test at the potentials just before OER onset, showing redox peaks of the cobalt ions.

PEIS analysis confirmed that the PL-fresh-3000 sample exhibited the smallest semicircle, indicating the lowest charge transfer resistance compared to the best catalysts from each series and the Co(OH)2 (Figure S3). The initial stability assessment was performed by comparing LSV curves recorded before and after chronoamperometric activity evaluation (Figure 5A). Reference Co(OH)2 decreased its activity, while the GNP-supported samples maintained high performance. The cyclic voltammetry of cobalt oxidation–reduction peaks shows the accessibility of reducible cobalt ions (Figure 5B), roughly following the cobalt content in the samples. The samples with the lowest cobalt content, prepared from 500 mg·dm^−3^ precursor concentrations, have the lowest redox peaks, while as the cobalt content in the samples increases, so does the redox peak intensity. The cobalt content quantification based on the XRF analysis, CVs and XPS analysis for the selected samples is presented in Table 1. The complementary data for all the catalysts are collected and presented in Table S3, and complete sets of CVs for all the samples are in Figure S4 in Supplementary Materials. For PL-fresh-3000 and PL-aged-3000 in Table 1, it can be noted that despite the same concentration of cobalt in these two samples measured by XRF, about 2.5 times more cobalt is available in PL-fresh-3000 for OER than PL-aged-3000. A higher cobalt content was determined with XPS for the PL-aged-3000 sample than for the other “3000” samples, which may be caused by a better cobalt phase dispersion as shown in Figure S1M,N in the SI.

The double-layer capacitance of the non-modified GNP was 143 μF, and it increased upon plasma oxidation. *C*_DL_ of the PL-fresh sample increased to 204 μF and decreased to 135 μF for the sample rinsed with acetate buffer after plasma treatment and before electrochemical measurements, PL-fresh-buffer. The values for the aged plasma-treated samples were 315 and 196 μF for PL-aged and GNP-PL-aged-buffer, respectively. This evaluation shows that the deposition of cobalt hydroxide and contacting plasma-treated GNP with the solution effectively suppresses the capacitive properties of the surface oxygen groups. Co(OH)_2_ was determined to exhibit a *C*_DL_ value equal to 1040 μF. In the composite samples, the C_DL_ values oscillated about 500 μF for the samples obtained from 500 mg·dm^−3^ Co^2+^ solution, then rose to about 1000 μF for intermediate samples, reaching a maximum in each series for the 3000 mg·dm^−3^ Co^2+^ solution, where C_DL_ reached 1400 μF for the 3000 (deposited over non-modified GNP) and PL-fresh-3000 samples. The full set of data is collected in Table S3.

**Figure 6:**
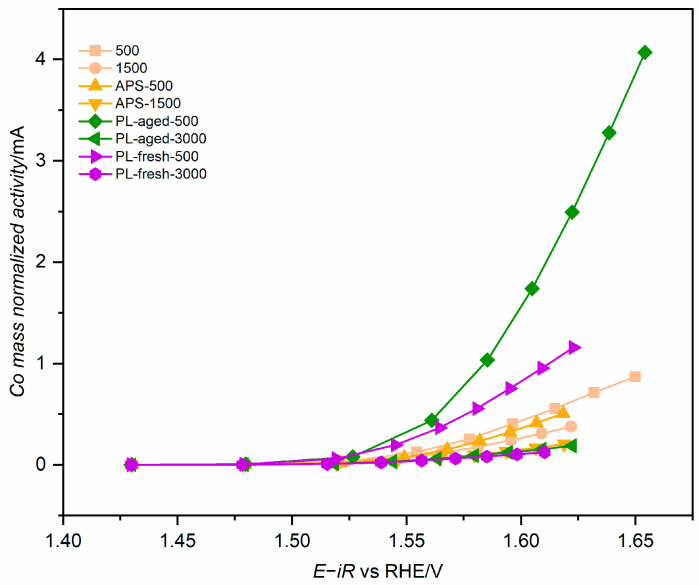
Mass-normalised OER activity of selected samples from each GNP support modification series.

The mass-normalised activity of the best samples and the samples with the lowest cobalt loading from each series is presented in Figure 6. The highest mass-normalised OER activity for each series is observed for the lowest cobalt loading. This indicates that the more cobalt in the composite, the less effective it is in OER. Microscopic analysis revealed that the cobalt active phase is present as agglomerates. Thus, much of the material is in poor contact with the conductive GNP support. The PL-aged-500 sample stands out in Figure 6 despite its low area-normalised activity because of the very low cobalt loading in this sample. Among the most competitive samples exhibiting overpotential below 330 mV, the PL-fresh-500 sample is the best-performing.

**Figure 7:**
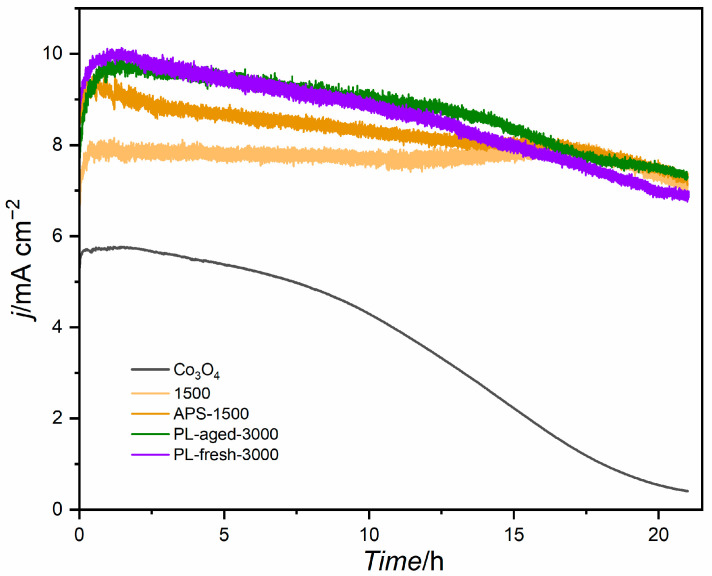
Stability tests of the cobalt spinel and best catalysts from each series at 1.65 V vs. RHE.

The preliminary LSV analysis before and after chronoamperometric activity testing indicated stable activity. Figure 7 shows the results of electrochemical stability tests of the best samples in each group of catalysts compared with cobalt spinel upon oxidation at 1.65 V vs. RHE for over 20 h. A decrease in the activity of the Co_3_O_4_ samples can be attributed to the degradation of the Nafion film containing the active phase since cobalt spinel is well-known for its stability in alkaline electrolytes. Despite the initially higher activity of the catalysts with plasma-modified GNP, their activity decreases over time. A similar trend can be observed for the APS-oxidised sample. Interestingly, the steadiest activity exhibits electrocatalysts obtained with non-modified GNP. In analysing the activity and stability results, it has to be remembered that a straightforward precipitation–deposition method has been used to obtain the studied materials. The reason was to obtain a cobalt hydroxide phase, which otherwise would transform into Co_3_O_4_ upon thermal treatment. Similarly, using organic molecules to improve the dispersion of the active phase would introduce additional components into the system, which would blur the analysis.

## 3. Materials and Methods

### 3.1. GNP Plasma Functionalisation

Graphene nanoplatelets (GNPs) with an average diameter of 30 μm, a surface area of 170 m^2^∙g^−1^, a thickness of 5 nm, and a conductivity in the range of 1.1 and 1.6∙103 S∙m^−1^ were purchased from Nanografi Nano Technology, Jena, Germany.

After grinding GNP in an agate mortar for about 10 min to obtain a soft powder, the powder was introduced to a low-temperature plasma in a plasma generator (Femto, Diener, Ebhausen, Germany) and synthetic air (Air Products, Krakow, Poland X40S COM, 2.2) for 5 min at 0.3 mbar pressure and 100 W generator power. The catalyst samples prepared using freshly oxidised GNP (just after plasma oxidation) are designated “GNP-PL-fresh”. Furthermore, to investigate the effect of plasma ageing on our catalysts, we kept air-plasma-oxidised GNPs in the lab cupboard for 14 days, designated “GNP-PL-aged” samples. For wet oxidation, 1 M ammonium persulfate (APS) solution was prepared in 2 M H_2_SO_4_. Then, 0.5 mg GNP was added to 30 mL of the APS solution in a round flask. The mixture was stirred and refluxed at 60 °C for 12 h. Then, the solid was filtered off and rinsed with distilled water and ethanol several times. The sample was dried in an oven at 60 °C for 12 h. Finally, oxygen plasma with pure oxygen (Air Products, Krakow, Poland, 99.9998% O_2_) for 5 min at 0.2 mbar pressure and 100 W was used, and the sample was named “GNP-APS”.

### 3.2. Catalyst Synthesis

The studied catalytic materials were prepared using a simple deposition–precipitation method, which involved alkalising the suspension of GNP with Co^2+^ ions using KOH solution. Four catalyst series were studied with different GNP as supports: non-modified GNP, GNP-APS, GNP-PL-fresh, and GNP-PL-aged. Samples were prepared using various concentrations of cobalt (II) perchlorate (Sigma-Aldrich, Saint Louis, MO, USA): 500, 1000, 1500, 2000 and 3000 mg·dm^−3^. An acetate buffer pH 5 with 0.1 M concentration was prepared and used as a solvent for cobalt solutions. For each sample preparation, 20 mg of oxidised GNP was mixed with 40 mL of cobalt solution in a shaker at 100 rpm for 24 h. This synthesis stage was introduced to ensure the equilibrium interaction of cobalt ions with the GNP as in the typical adsorption studies. Then, the mixture was titrated with 0.1 M KOH, and the precipitation was filtered off and dried in an oven at 60 °C for 24 h. Cobalt solution concentrations and air plasma conditions were constant for all catalyst series. Sample names were designated to reflect the preparation history but also to be simple; thus, the GNP does not appear in their names. Thus, non-modified GNP-based catalysts are designated only with the cobalt precursor concentration xxxx, where xxxx stands for 500, 1000, 1500, 2000 and 3000 mg·dm^−3^, e.g., “1500”. GNP-APS-based samples are named APS-xxxx, while GNP-PL-fresh-based and GNP-PL-aged-based samples are named PL-fresh-xxxx and PL-aged-xxxx, respectively.

### 3.3. Physicochemical Characterisation

An SESR4000 analyser (Gammadata Scienta, Uppsala, Sweden) was applied to take X-ray photoelectron spectra in a vacuum with a base pressure below 5∙10^−9^ mbar. A monochromatic Al-Kα source with 250 W at 1486.6 eV emission energy was utilised, and the pass energy for selected narrow binding energy scans was 100 eV. The CASA XPS programme was used to analyse the X-ray photoelectron spectroscopy (XPS) spectra with the built-in quantitative analysis feature [23]. The C 1s binding energy range was assayed at 292–280 eV. The energy scale was calibrated by applying the gold work function of 4.65 eV. A Renishaw InVia spectroscope with a 514.5 nm laser and a Leica microscope was used to record Raman spectra. X-ray fluorescence spectroscopy (XRF) utilising the Thermo Scientific ARL QUANT’X spectrometer with UniQuant ED 6.14 software determined the cobalt content in the samples. Scanning electron microscopy (SEM) images were taken using a ThermoFisher Scientific Helios 5 Hydra CX PFIB microscope (Waltham, MA, USA). Images were collected with active immersion optics and the through-the-lens detector in secondary electrons (SE) and backscattered electrons (BSE) mode. The accelerating voltage was set at 10 kV. Samples were placed on carbon tape as received to obtain proper fixation and electric contact.

### 3.4. Electrocatalytic Test

All the electrochemical measurements were recorded using a Biologic BP-300 connected to a Biologic RC-10K (Seyssinet-Pariset, France). Catalyst ink was deposited on a glassy carbon electrode (GCE) surface with a 3 mm diameter to obtain 200 µg·cm^−2^ loading. GCE was used as a rotating disc electrode (RDE) at 1600 rpm during the measurements. A three-electrode cell was assembled, including RDE, a Hg/HgO in NaOH 1.0 mol L^−1^ as a reference electrode and a platinum wire as an auxiliary electrode, immersed in 50 mL KOH 0.1 M solution as an electrolyte. The electrolyte solution was saturated with argon gas for 45 min before starting every measurement, and the cell was flushed with it during the experiment. Before starting every measurement, the GCE surface was polished mechanically by using an aluminium oxide slurry (0.05 µm). Then, it was left in the ultrasound bath for 2–3 min to remove the residual Al_2_O_3_. All the applied potentials were recalculated to the potential of the reversible hydrogen electrode (RHE) by using the *E*_RHE_ = *E*_HgO/Hg_^Θ^ + *E*_Hg/HgO_ + 0.059 pH equation, where E_HgO/Hg_^Θ^ = 0.098 V vs. NHE at T = 25 °C. Electrolyte pH was measured by using a pH metre before starting every measurement.

Catalyst ink was prepared by dispersing 1.9 mg of finely ground catalyst in 375 µL distilled water, 125 µL isopropyl alcohol and 25 µL Nafion (5% solution) in the ultrasound bath for 30 min. Furthermore, 3.9 µL of ink was deposited on the GCE surface and dried for 45 min at 200 rpm to obtain a thin film layer (TFL) of the catalyst. The obtained catalyst loading on GCE was 200 µg·cm^−2^. We observed poor stability during stability tests, which we ascribed to the TFL degradation. Thus, we optimised the ink composition used to evaluate the catalyst’s stability by decreasing the amount of Nafion as a binder since we found that Nafion decomposes upon long-time oxidation, affecting the catalyst’s TFL stability. Therefore, 2 mg of every catalyst powder was dispersed in 250 µL distilled water, 750 µL isopropyl alcohol and only 5 µL of Nafion (5% solution) in the ultrasound bath for 30 min. Then, 7.1 µL of ink was deposited dropwise on GCE to obtain 200 µg·cm^−2^ loading and dried as described above.

Electrochemical measurements were started by stabilising the TFL of catalysts by scanning potentials between 0.2 and 0.9 V vs. RHE by using the cyclic voltammetry technique (CV) with 10 cycles at 100 mV/s scan rate, 10 cycles at 20 mV/s scan rate and 5 cycles at 10 mVs^−1^ scan rate. Then, another series of CVs were applied at 1.2–1.3 V vs. RHE at 2, 4, 6, 8, 10 and 12 mVs^−1^ (3 cycles in each scan rate) to calculate double-layer capacitance (*C*_DL_). CVs were also scanned 5 times at 0–0.635 V vs. Hg/HgO to record cobalt redox peaks. Chronoamperometry (CA) was applied in 9 potential steps from 1.43 V vs. RHE to 1.83 V vs. RHE with a 50 mV increment between the steps and by holding every potential step for 15 min as the primary technique of evaluating catalyst activity. The ohmic drop was measured before and after CA by applying potentiostatic electrochemical impedance spectroscopy (PEIS) at 1.55 V vs. RHE. Furthermore, linear sweep voltammetry (LSV) was applied for one scan at 1.23–1.6 V vs. RHE before and after CA to see any changes in electrochemical activity by oxidation of TFL at 1.83 V vs. RHE (last CA step). A preliminary stability test was applied at the end of catalyst activity measurements by recording 10 cycles of CVs between 0.865 and 2 V vs. RHE. The stability test was recorded at 1.65 V vs. RHE by applying the CA technique for 21 h.

## 4. Conclusions

In this study, we aimed to investigate whether the OER activity of GNP-supported cobalt hydroxide depends on the carbon support pretreatment. We discovered that the obtained materials are competitive with Co_3_O_4_-based electrocatalysts regarding OER activity and exhibit improved stability compared to a reference Co_3_O_4_ material. The dispersion of the active phase did not change for different pretreatment methods; thus, some synergistic effects with the GNP support can be hypothesised, the nature of which should be further studied. Based on the XPS data, we conclude that the enhanced interaction of the active phase with the support is due to the plasma-activated carbon surface, resulting in an increased ratio of surface oxygen groups and their distinct speciation (increased C=O, carbonyl groups and carbons attached to two ether/hydroxyl groups). The results indicate that plasma activation of GNP support maximises the usage of the transition metal active phase, which allows for an improvement in area-normalised and a dramatic improvement in the mass-normalised OER electrocatalytic activity.

## Figures and Tables

**Table 1:** Cobalt content of our chosen catalysts based on CVs and XRF measurements compared with catalyst activity based on overpotentials formed @10 mA·cm^−2^.

Sample	Co Cont./µmole Peak_ox_	Co wt.% XRF	Co at.% XPS	C_DL_/µF	Tafel Slope/Dec^−1^
500	1.80 × 10^−3^	1.6	-	472	46
1500	8.14 × 10^−3^	9.5	10.6	1100	43
APS-500	3.46 × 10^−3^	5.4	-	545	42
APS-1500	4.90 × 10^−3^	13.3	3.9	1200	45
PL-aged-500	9.48 × 10^−4^	0.5	-	466	47
PL-aged-3000	5.01 × 10^−3^	22.5	20.5	1030	40
PL-fresh-500	8.17 × 10^−4^	2.4	-	515	44
PL-fresh-3000	1.25 × 10^−2^	22.6	9.3	1400	43

## Data Availability

No data is provided with the manuscript.

## Supplementary Materials

The following supporting information can be downloaded at: https://www.mdpi.com/article/10.3390/molecules30071522/s1. Table S1: Surface oxygen quantification with XPS of the modified GNP samples; Table S2: XPS quantification of the Co-modified GNP samples; Table S3: Numeric values of materials characterisation, double layer capacitance values, cobalt content, the overpotentials@10mAcm-2 for all the catalysts and the references studied; Figure S1: SEM images in BSE mode of A) GNP, B) GNP-APS, C) and D) GNP-PL-aged, E) and ) PL-fresh-1500, G: PL-aged-1500, H) PL-fresh-3000, I) PL-aged-3000, J) 1500, K) and L) APS-3000. SEM images in SE mode of M) PL-aged-3000, N) PL-fresh-3000; Figure S2: iR-corrected summary of CA measurements for reference GNP, cobalt hydroxide, and the best catalyst of each series; Figure S3: PEIS results recorded before CA, for the best catalyst of each series and cobalt hydroxide; Figure S4: CVs recorded just before CA for each series of catalysts.
