# Supplementary material for: Maximising the Potential of Reactive Carbon Support with Cobalt Active Phase for the Oxygen Evolution Reaction
Source: Molecules. 2025 Mar 29;30(7):1522. doi: 10.3390/molecules30071522 (PMC11990261; doi:10.3390/molecules30071522)
Supplement: Supplementary file 1 [file molecules-30-01522-s001.zip › molecules-3561873-supplementary.pdf]

Supporting Information

for

Maximising the Potential of Reactive Carbon Support with Cobalt Active Phase for the Oxygen Evolution Reaction

*Termeh Darvishzad[1], Paweł Stelmachowski[1]**

[1]Jagiellonian University, Faculty of Chemistry, Gronostajowa 2, 30-387 Krakow, Poland

*pawel.stelmachowski@uj.edu.pl

Table S1.Surface oxygen quantification with XPS of the modified GNP samples.

Sample	Component	at.%
GNP	O 1s	2
	C 1s	98
GNP-PL-fresh	O 1s	12
	C 1s	88
GNP-PL-aged	O 1s	8
	C 1s	92
GNP-PL-aged-buffer	O 1s	6
	C 1s	94
GNP-PL-fresh-buffer	O 1s	7
	C 1s	93
GNP-APS	O 1s	6
	C 1s	94

Table S2. XPS quantification of the Co-modified GNP samples.

Sample	Component	at.%
PL-fresh-1500	Co 2p	6
	O 1s	15
	C 1s	79
PL-fresh-3000	Co 2p	9
	O 1s	22
	C 1s	69
1000	Co 2p	8
	O 1s	13
	C 1s	79
1500	Co 2p	11
	O 1s	20
	C 1s	69
2000	Co 2p	9
	O 1s	17
	C 1s	74
3000	Co 2p	9
	O 1s	15
	C 1s	76
PL-aged-1000	Co 2p	8
	O 1s	15
	C 1s	77
PL-aged-1500	Co 2p	6
	O 1s	11
	C 1s	83
PL-aged-3000	Co 2p	20
	O 1s	34
	C 1s	46
APS-1500	Co 2p	4
	O 1s	10
	C 1s	86
APS-3000	Co 2p	7
	O 1s	16
	C 1s	77

Table S3. Numeric values of materials characterisation, double layer capacitance values, cobalt content, the overpotentials@10mAcm^{-2} for all the catalysts and the references studied.

Sample name	Double layer capacitance, C_{DL}/µF	Co mass (µmole) $peak_{ox}$	Co mass (µmole) $peak_{red}$	w_{Co3O4}/wt.% XRF	Activity ($\eta_{10\ mA.cm^{-2}}$/mV)
**500**	472	1.80E-03	1.71E-03	1.6	345
**1000**	960	6.12E-03	5.61E-03	3	331
**1500**	1100	8.14E-03	6.62E-03	9	328
**2000**	1020	3.76E-04	3.28E-04	20	338
**3000**	1400	1.07E-02	7.89E-03	23	333
**APS-500**	545	3.46E-03	2.96E-03	5	339
**APS-1000**	1000	3.55E-03	2.92E-03	11	331
**APS-1500**	1200	4.90E-03	4.24E-03	13	329
**APS-2000**	1200	1.07E-02	8.25E-03	13	338
**APS-3000**	1100	5.68E-03	4.18E-03	23	340
**PL-aged-500**	466	9.48E-04	9.42E-04	0.5	365
**PL-aged-1000**	575	1.16E-03	1.01E-03	2	355
**PL-aged-1500**	715	3.72E-03	3.16E-03	5	350
**PL-aged-2000**	700	4.13E-03	3.24E-03	10	342
**PL-aged-3000**	1030	5.01E-03	5.10E-03	22	325
**PL-fresh-500**	515	8.17E-04	9.52E-04	2	327
**PL-fresh-1000**	1020	9.49E-03	7.73E-03	7	326
**PL-fresh-1500**	1360	1.22E-02	9.39E-03	8	323
**PL-fresh-2000**	930	2.57E-03	2.31E-03	13	324
**PL-fresh-3000**	1400	1.25E-02	9.55E-03	23	317

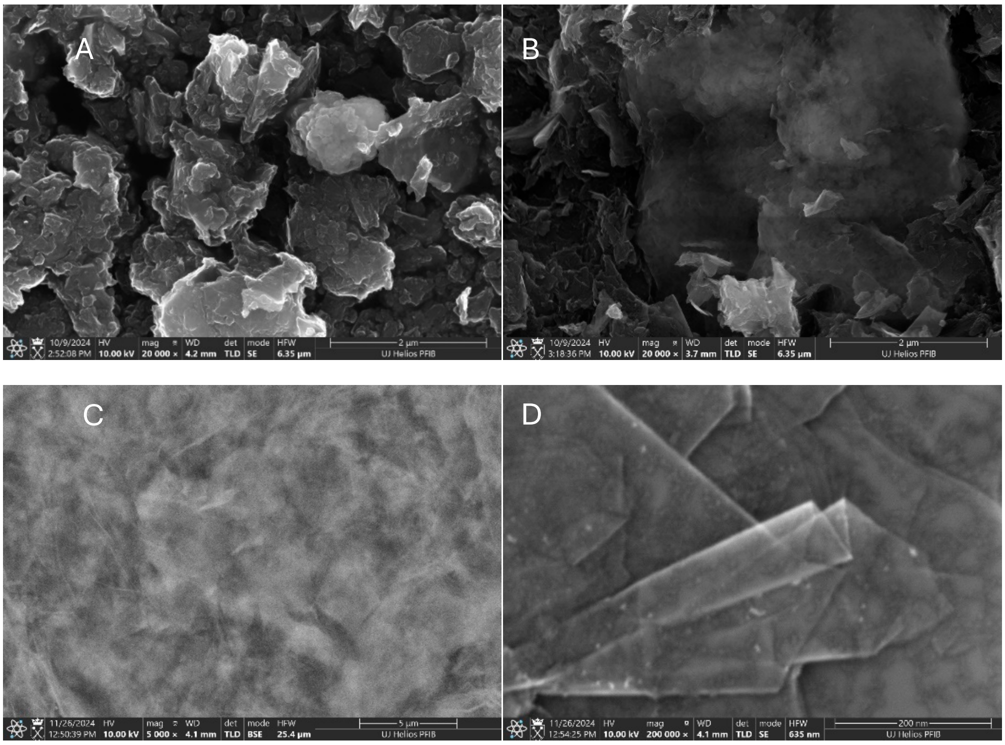
A
B
C
D

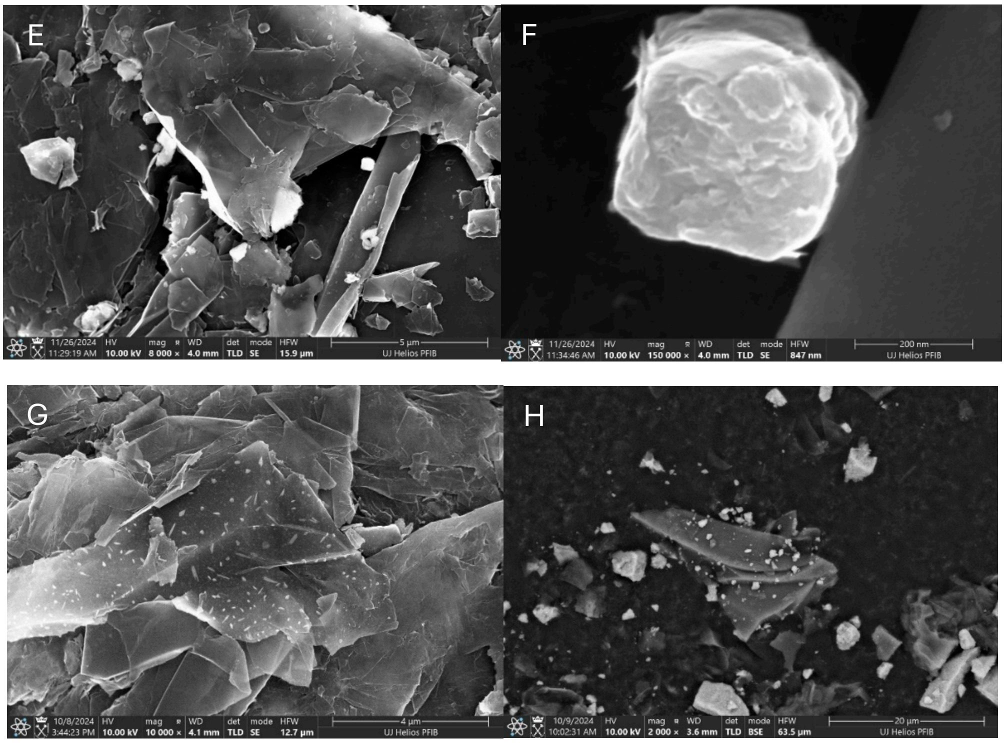
E
F
G
H

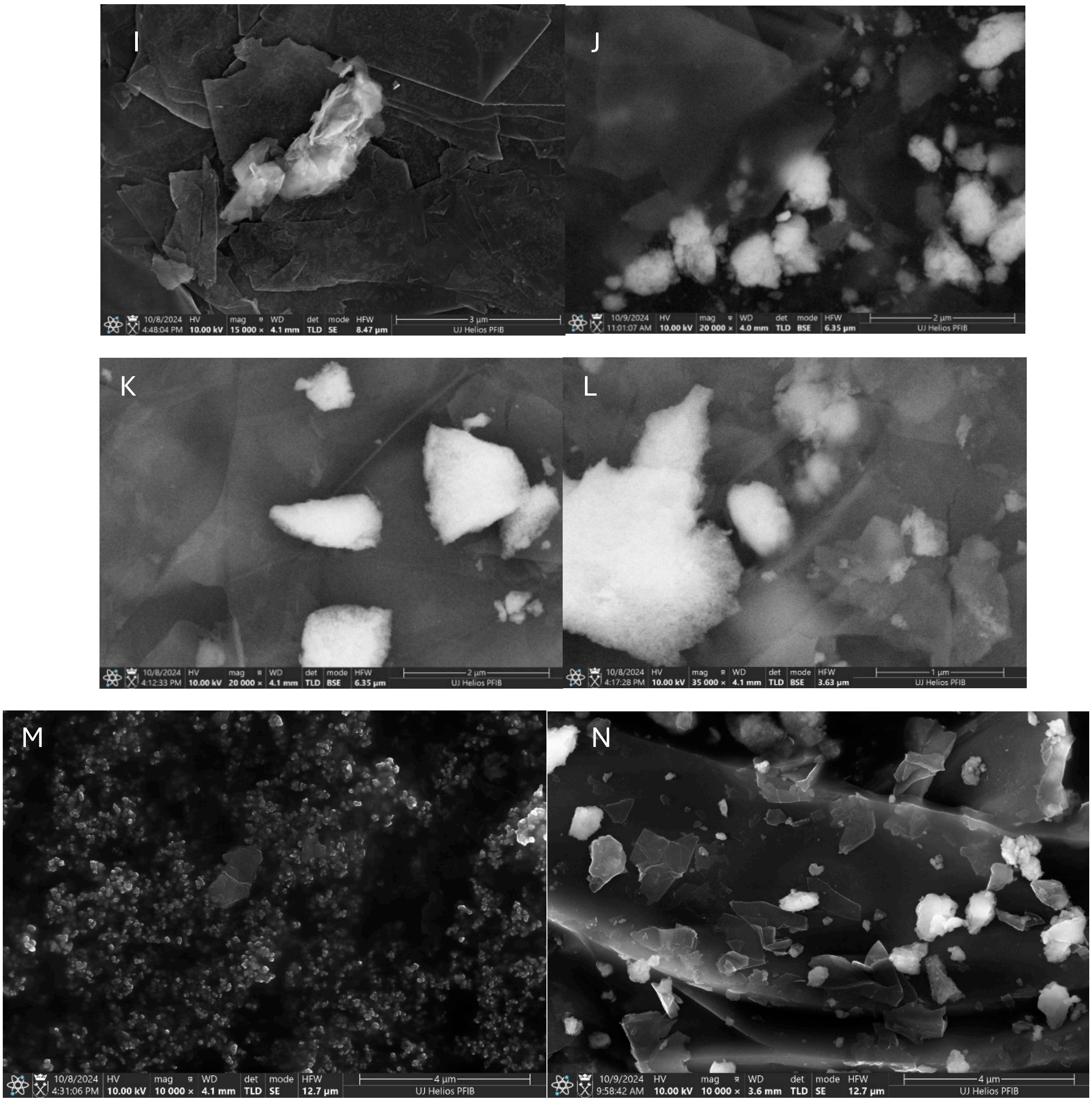

Figure S1. SEM images in BSE mode of A) GNP, B) GNP-APS, C) and D) GNP-PL-aged, E) and) PL-fresh-1500, G: PL-aged-1500, H) PL-fresh-3000, I) PL-aged-3000, J) 1500, K) and L) APS-3000. SEM images in SE mode of M) PL-aged-3000, N) PL-fresh-3000.

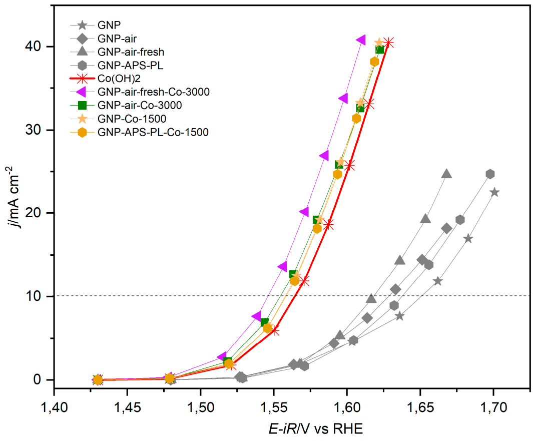

Figure S2: **iR-corrected summary of CA measurements for reference GNP, cobalt hydroxide, and the best catalyst of each series.**

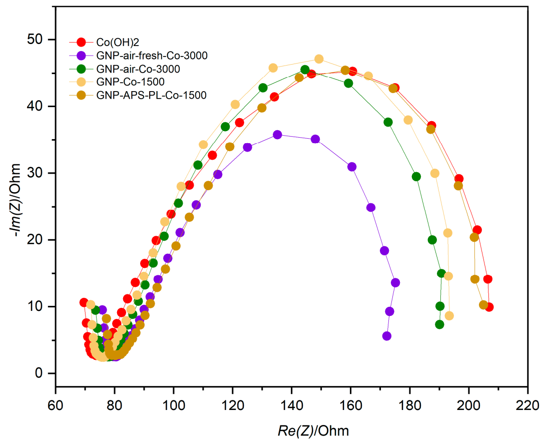

Figure S3: **PEIS results recorded before CA, for the best catalyst of each series and cobalt hydroxide.**

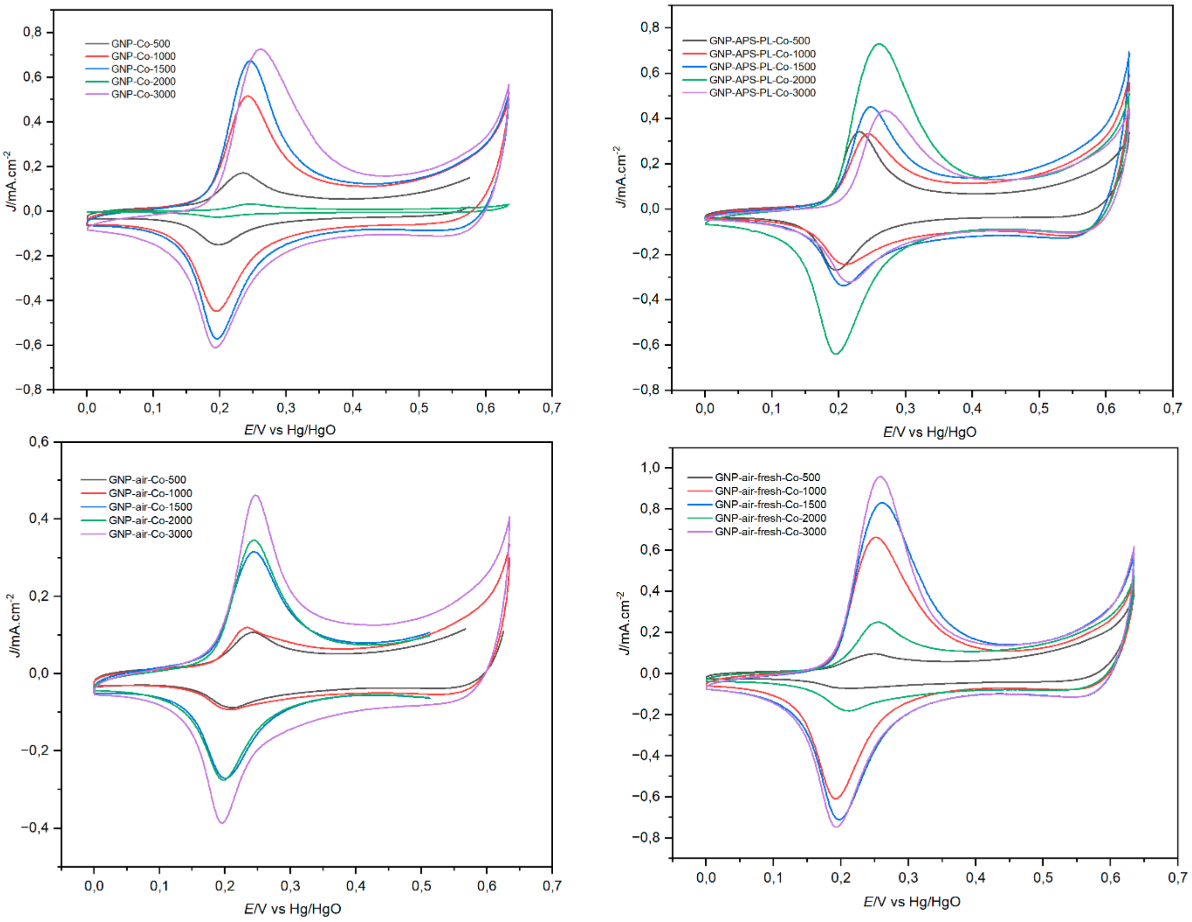

Figure S4: **CVs recorded just before CA for each series of catalysts.**
